# Complement activation in polycystic ovary syndrome occurs in the postprandial and fasted state and is influenced by obesity and insulin sensitivity

**DOI:** 10.1111/cen.14322

**Published:** 2020-09-15

**Authors:** Ruth D. Lewis, Anil K. Narayanaswamy, Daniel Farewell, Dafydd Aled Rees

**Affiliations:** ^1^ Division of Infection and Immunity School of Medicine Cardiff University Cardiff UK; ^2^ Division of Psychological Medicine and Clinical Neurosciences School of Medicine Cardiff University Cardiff UK; ^3^ Division of Population Medicine School of Medicine Cardiff University Cardiff UK

**Keywords:** complement system proteins, insulin resistance, obesity, polycystic ovary syndrome

## Abstract

**Objective:**

Polycystic ovary syndrome (PCOS) is associated with metabolic risk. Complement proteins regulate inflammation and lipid clearance but their role in PCOS‐associated metabolic risk is unclear. We sought to establish whether the complement system is activated in PCOS in the fasting and postprandial state.

**Design:**

Case‐control study.

**Patients:**

Fasting complement levels were measured in 84 women with PCOS and 95 healthy controls. Complement activation post‐oral fat tolerance test (OFTT) was compared in 40 additional subjects (20 PCOS, 20 controls).

**Measurements:**

Activation pathway (C3, C4, C3a(desArg), factor B, factor H, properdin, Factor D) and terminal pathway (C5, C5a, terminal complement complex [TCC]) proteins were measured by commercial or in‐house assays.

**Results:**

Fasting C3, C3a(desArg) and TCC concentrations were increased in insulin‐resistant (adjusted differences: C3 0.13 g/L [95%CI 0‐0.25]; C3a(desArg) 319.2 ng/mL [19.5‐619]; TCC 0.66 μg/mL [0.04‐1.28]) but not in insulin‐sensitive women with PCOS. C3 and factor H levels increased with obesity. Post‐OFTT, C3 and C4 levels increased to a similar extent in PCOS subjects and controls, whist factor H levels increased more in women with PCOS compared to controls (adjusted differences (area under the curve): 12 167 μg min/mL [4942‐19 392]), particularly in the presence of concomitant obesity.

**Conclusions:**

Activation and terminal complement pathway components are elevated in patients with PCOS, especially in the presence of insulin resistance and obesity.

## 1 INTRODUCTION

Polycystic ovary syndrome (PCOS) is a common endocrine disorder associated with hyperandrogenism, insulin resistance and dyslipidaemia. These disturbances lead to an increased risk of cardiometabolic disease including type 2 diabetes.^1^ The underlying drivers of this process are unclear although chronic inflammation may play an important role.^2^

The complement system is a key regulator of inflammation and consists of three activation pathways: classical, alternative and lectin, which converge at the level of C3 to form C3 convertases (Figure S1). Whilst the classical and lectin pathway convertases depend on C2 and C4 cleavage, the alternative pathway convertase requires factor B and factor D. Further activation of the complement system through to the terminal pathway involves C5 cleavage and leads to formation of the membrane attack complex and its fluid‐phase by‐product, the terminal complement complex (TCC). Both positive and negative regulators exist, including the alternative pathway regulators properdin and factor H.

Components of the complement system, notably C3, have been shown to be increased in patients with metabolic syndrome,^3^ type 2 diabetes^4^ and cardiovascular disease.^5^ Postprandially, C3 activation has been shown to increase lipid clearance and storage in human adipocytes^6^, ^7^; C3a, a product of C3 activation, is rapidly cleaved in plasma to form C3a(desArg). C3a(desArg) binds to its receptor, C5L2, on adipocytes to increase triglyceride synthesis.^6^, ^8^, ^9^ Chylomicrons, transporters of lipids in the postprandial period, have been shown to increase C3 activation,^10^ an event that is regulated in vivo by factor H.^11^ Furthermore, activation pathway components (C3, C4, factor D and factor B) alter after a meal, and postprandial C3 responses differ in patients with and without cardiometabolic disease.^12^, ^13^, ^14^, ^15^ These findings suggest that dysregulated postprandial complement activation may influence metabolic health and contribute to the development of cardiometabolic pathology.

Only a few studies have examined the complement system in women with PCOS, finding increased levels of factor D^16^ and C3a(desArg),^17^, ^18^ and increased^18^, ^19^ or no difference^20^, ^21^ in C3 levels compared to matched controls. However, many of these studies are limited by fasting measurements only and small sample sizes. We therefore sought to establish whether the complement system is activated in women with PCOS and whether any abnormalities are evident in the postprandial as well as the fasting state.

## 2 MATERIALS AND METHODS

### 2.1 Overall study design

We undertook the study in two parts. We firstly compared fasting plasma complement protein levels between insulin‐resistant patients with PCOS (n = 84) and healthy controls (n = 95; cohort 1). Plasma samples, maintained at −80°C, were obtained from our previous study in which detailed anthropometric, metabolic and cardiovascular phenotyping was undertaken.^22^ We then compared fasting and post‐oral fat tolerance test (OFTT) complement levels in PCOS women (n = 20) and healthy controls (n = 20; cohort 2), in order to determine any contribution of postprandial lipaemia to complement activation.

### 2.2 Inclusion and exclusion criteria

For both cohorts, patients with PCOS (aged 16‐45 years) were recruited from a departmental database or from outpatients attending the University Hospital of Wales. A diagnosis of PCOS was made according to the Rotterdam criteria, with congenital adrenal hyperplasia, androgen‐secreting tumours, Cushing's syndrome, thyroid disease and hyperprolactinaemia excluded by biochemical testing. Subjects were also excluded if they were pregnant, breastfeeding or had a history of hypertension, hyperlipidaemia or diabetes, or current or previous (within 3 months) use of glucocorticoids, lipid‐lowering agents, antihypertensives, antidiabetics or anti‐obesity drugs. Healthy volunteers were recruited by advertisement in the local press, and among students and staff within our Institution. Healthy volunteers had regular menstrual cycles (every 27‐32 days) and their healthy state was established by history, physical examination and hormonal evaluation (thyroid function, prolactin, testosterone and 17‐hydroxyprogesterone); those with features of hirsutism or a family history of PCOS were excluded. For cohort 1, in the PCOS group there were 15 current smokers and 12 ex‐smokers (32.1%) compared with 11 current smokers and 19 ex‐smokers among the healthy volunteers (31.6%). Nineteen subjects with PCOS (22.6%) were taking a combined oral contraceptive pill compared with 28 (29.5%) healthy volunteers. For cohort 2, in the PCOS group there were two current smokers and one ex‐smoker (15%) compared with one current smoker and two ex‐smokers among the healthy volunteers (15%). Of the PCOS group, 8 (40%) were taking a combined oral contraceptive pill compared with 7 (35%) healthy volunteers. The study was approved by Cardiff and Vale University Health Board, Cardiff University and the South East Wales Research Ethics committee (reference number 08/WSE/04/53). All subjects gave written informed consent prior to study commencement.

### 2.3 Postprandial study protocol and assessment of insulin sensitivity

Cohort 2 subjects attended our Clinical Research Facility at 09:00 hours after an overnight fast. The standard OFTT meal comprised fresh cream with 40% (weight by volume; w/v) fat emulsion (polyunsaturated:saturated fat ratio of 0.10) that contained 0.001% (w/v) cholesterol and 3% (w/v) carbohydrates and had a total energy content of 3700 kcal/L.^13^ The fresh cream was given at a dose of 50 g fat and 3.75 g glucose/m^2^ body surface (approximately 200 mL). Subjects were allowed up to 10 minutes to consume the meal. During the test, participants remained supine and were only allowed to drink mineral water. Blood samples were obtained at 0 (fasting baseline), 30, 60, 120, 180 and 240 minutes after consuming the meal, collected into sodium EDTA (2 mg/mL) then centrifuged immediately for 15 minutes at 800 *g* at 4°C. Plasma was separated and stored in aliquots at −80°C until analysis. The areas under the curves (AUC) for triglycerides and complement components were calculated using the trapezoid method.

On a separate day, after an overnight fast, subjects in cohort 2 underwent basal sampling for measurement of lipids and testosterone. Subjects subsequently underwent a standard 75 g oral glucose tolerance test. Glucose and insulin were measured at 0, 30, 60, 90 and 120 minutes. The AUCs for insulin and glucose were calculated using the trapezoid method. The homeostatic model assessment method was also used to estimate insulin resistance (HOMA‐IR).

### 2.4 Anthropometric measurements

Height, weight, waist and hip circumference were measured according to our published protocol.^23^ Abdominal subcutaneous (SF) and visceral (VF) fat areas were measured by X‐ray computed tomography (CT; Hawkeye, GE Medical Systems) as previously described.^23^ CT images were segmented into fat and nonfat areas according to our previously published protocol.^23^

### 2.5 Biochemical analysis

Plasma total cholesterol, HDL and triglyceride levels were measured using an Aeroset automated analyser (Abbott Diagnostics). Insulin levels were assessed using an immunometric assay specific for human insulin (Invitron), and glucose was measured using the Aeroset chemistry system (Abbott Diagnostics). Total testosterone was measured by liquid chromatography‐tandem mass spectrometry (Quattro™ Premier XE triple quadrupole tandem mass spectrometer; Waters Ltd). Plasma C3 and C4 levels were quantified by nephelometry on a Beckman BN11 nephelometer in the University Hospital of Wales Clinical Immunology laboratory using commercial standards. The assay working range for C3 was 0.02‐4.1 g/L, and for C4 was 0.01‐1.9 g/L. C5a(desArg), C3a(desArg), factor D and properdin were quantified using their respective commercial assays from Hycult Biotech, as instructed by the manufacturer. Plasma C5, TCC and factor H were all measured using in‐house ELISA.^24^, ^25^, ^26^ All assays used purified protein (either C5, TCC or factor H) as a standard. For C5 ELISA, plates were coated with an in‐house polyclonal rabbit antihuman C5 antibody (8 μg/mL, 100 µL/well, in bicarbonate buffer [pH 9.6]), blocked in 2% (w/v) bovine serum albumin (BSA; blocking buffer), and then incubated with plasma samples diluted one in 600 in blocking buffer. Mouse monoclonal antihuman C5 (MBI‐C5‐3; 5 μg/mL) was used to detect bound C5, followed by donkey antimouse IgG horseradish peroxidase (HRP; 1 in 2500).

For TCC quantification, plates were coated with an in‐house anti‐C9 neo‐antibody (B7), used at 4 µg/mL, 100 µL/well. After blocking with 2% (w/v) BSA, plasma samples were diluted 1:6 in blocking buffer. HRP‐conjugated monoclonal mouse antihuman C8 (clone E2, in‐house) was added to wells for detection of TCC (100 µL; 2 µg/mL). For total factor H ELISA, plates were coated with affinity‐purified rabbit antifactor H IgG diluted in bicarbonate coating buffer (pH 9.6) at 5 μg/well. After blocking with 1% (w/v) BSA, plasma samples were diluted 1:6000 in blocking buffer. HRP‐labelled affinity‐purified rabbit antihuman factor H (100 µL; 1 mg/L) was used to detect total factor H. Of note, many papers quote higher levels of serum factor H; the extinction coefficient for factor H standards on which our normal range is based has been validated previously.^26^

### 2.6 Statistical analysis

Linear regression models were used to assess differences in mean biomarker levels between PCOS and control groups, adjusting for age, BMI and smoking wherever the latter were found to have explanatory value. Best fitting models were selected on the basis of Akaike's information criterion (AIC). A two‐tailed *P*‐value of <.05 was considered significant. All analyses were performed with the GraphPad Prism version 5 Windows and the R language and environment for statistical computing.

## 3 RESULTS

### 3.1 Fasting complement concentrations in insulin‐resistant PCOS subjects and healthy controls

The clinical, metabolic and anthropometric characteristics of cohort 1 are shown in Table SI1. As anticipated, PCOS women had higher androgen levels and worse insulin sensitivity, even after adjustment for age and BMI. Table 1 shows the concentration of plasma complement components and activation products in PCOS subjects and controls, before and after adjustment. Plasma C3, C3a(desArg), C3a(desArg)/C3 ratio and TCC levels were significantly increased in the PCOS group compared to controls, even after adjustment. Factor B, factor H and factor D were all significantly increased in PCOS before, but not after, adjustment for BMI, age and smoking. Conversely, properdin was significantly increased in the PCOS group but only after adjusting for age, BMI and smoking. Fasting levels of C3, C3a(desArg), C4, FH and TCC were unaffected by oral contraceptive status (data not shown).

**Table 1:** Fasting plasma complement levels in cohort 1

	PCOS	Control	Unadjusted				Adjusted for age, BMI and smoking			
			2.5% CI	Difference	97.5% CI	*P*‐value	2.5% CI	Bestfit	97.5% CI	*P*‐value
C4 (g/L)	0.45 (−0.21)	0.37 (−0.19)	0.01	0.08	0.15	.03	−0.05	0.01	0.08	.67
C3 (g/L)	1.67 (−0.39)	1.4 (−0.36)	0.14	0.27	0.41	<.001	<0.0001	0.13	0.25	.04
C3a(desArg) (ng/mL)	687.67 (−816.21)	373.81 (−500.73)	19.01	313.86	608.71	.04	19.5	319.23	618.96	.04
C3a/C3 ratio	924.01 (−873.54)	485.33 (−713.34)	106.7	438.68	770.67	.01	149.25	506.76	864.26	.01
FB (μg/mL)	140.34 (−36.14)	125.12 (−33.43)	1.41	15.22	29.03	.03	−12.89	1.52	15.94	.83
FH (μg/mL)	183.33 (−50.44)	163.52 (46.98)	3.41	19.81	36.21	.02	−15.44	−1.32	12.79	.85
Properdin (μg/mL)	15.31 (−4.18)	14.58 (3.98)	−0.68	0.73	2.15	.31	−2.64	−1.32	−0.01	.05
FD (μg/mL)	0.84 (−0.21)	0.78 (0.16)	<0.0001	0.07	0.13	.04	−0.04	0.03	0.09	.41
C5a (μg/mL)	18.31 (−22.95)	25.39 (23.36)	−16.85	−7.07	2.71	.15	−15.6	−5.76	4.08	.25
C5 (μg/mL)	183.76 (−57.04)	167.23 (55.27)	−5.32	16.53	38.37	.14	−17.29	5.75	28.79	.62
TCC (μg/mL)	3.55 (−1.72)	2.94 (1.82)	<0.0001	0.61	1.23	.05	0.04	0.66	1.28	.04

Note

Data are presented as means (± standard deviation). n = 84 PCOS, n = 95 Controls.

Abbreviations: CI, confidence interval; FB, Factor B; FH, Factor H; FD, Factor D; TCC, Terminal complement complex.

**Figure 1:**
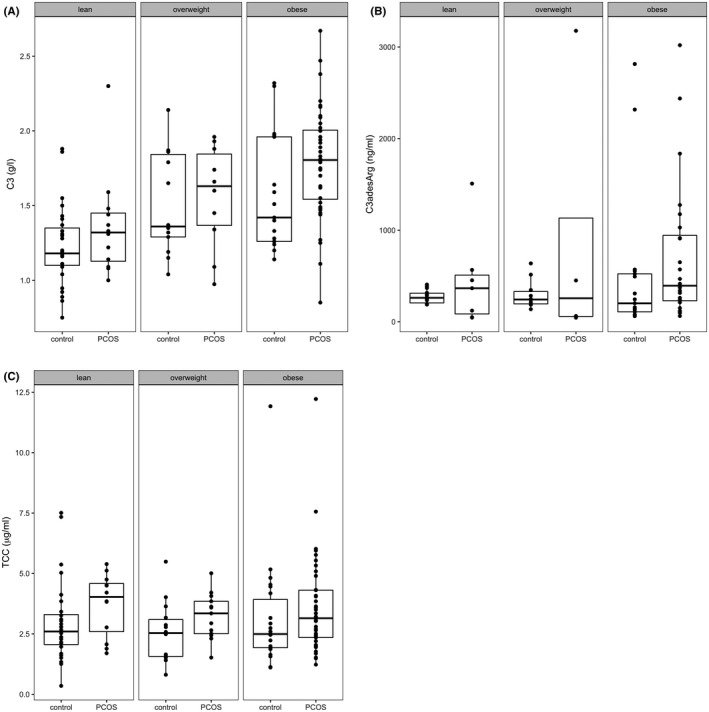
Influence of BMI on fasting C3, C3adesArg and TCC concentrations in PCOS and control subjects (cohort 1). Boxplots of C3 (A), C3adesArg (B) and TCC levels in PCOS subjects and metabolically healthy controls. Data grouped according to BMI (lean: BMI 18.5‐24.99; overweight: BMI 25‐29.99 and obese: BMI ≥ 30 kg/m^2^). Middle lines show the medians of the data and the boxes highlight the 25th and 75th percentiles (Quarter 1 and Quarter 3). C3 levels were significantly higher in obese subjects with PCOS compared to obese controls (mean difference ± SEM; 0.222 ± 0.099 g/L, *P* < .05). TCC: terminal complement complex. n = 84 PCOS, n = 95 Controls

Figure 1 shows the fasting plasma concentrations of C3, C3a(desArg) and TCC in PCOS subjects and healthy controls, stratified according to BMI. C3 levels increased across BMI categories in both groups but between‐group differences were only apparent in obesity (mean difference ± SEM; 0.22 ± 0.1 g/L; *P* < .05). In contrast, C3a(desArg) and TCC levels were not affected by BMI.

### 3.2 Associations of fasting complement concentrations and metabolic parameters

The associations between circulating complement levels and a range of anthropometric and metabolic risk measures are shown in Table S2. Across the whole cohort (PCOS and controls), triglycerides, and to a lesser extent LDL cholesterol, correlated strongly with early complement pathway components and activation products but weakly with terminal components and activation products (C5, C5a(desArg) and TCC). Both early and late complement pathway components were significantly associated with both visceral and subcutaneous fat area. HOMA‐IR was most strongly associated with C3, factor H and properdin.

### 3.3 Fasting complement concentrations in insulin‐sensitive PCOS subjects and healthy controls

Table S3 summarizes the clinical and metabolic characteristics of the PCOS and healthy control groups in cohort 2. PCOS and healthy volunteers were well matched for age and BMI. As expected, testosterone concentrations were increased in PCOS subjects compared with controls but measures of insulin resistance (insulin AUC and HOMA‐IR) were not different between groups. Furthermore, in contrast to cohort 1, there were no differences in fasting complement concentrations between groups (Table S4).

### 3.4 Effect of postprandial lipaemia on plasma complement concentrations in PCOS subjects and controls

**Figure 2:**
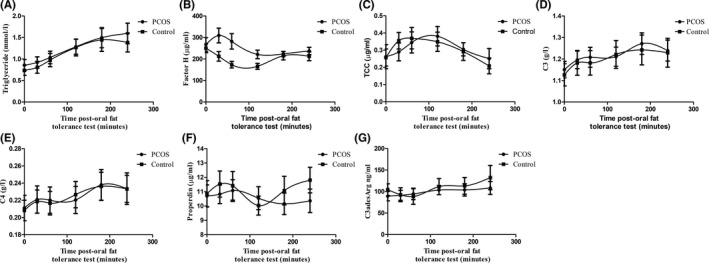
Effects of postprandial lipaemia on triglyceride and complement concentrations in PCOS and control subjects. Postprandial triglyceride (A), factor H (B), TCC (C), C3 (D), C4 (E), properdin (F) and C3adesArg (G) levels in response to OFTT in PCOS cohort and control groups (cohort 2). Data are mean ± SEM. n = 20 PCOS, n = 20 Controls

To compare the effects of postprandial lipaemia on complement secretion, PCOS subjects and healthy volunteers in cohort 2 underwent an OFTT. As expected, plasma triglyceride levels increased significantly from baseline in both PCOS and control groups (Figure 2A), reaching maximum levels (*T*_max_) at 240 minutes (mean ± SEM; 0.86 ± 0.13 mmol/L [baseline] vs 1.6 ± 0.24 mmol/L) and 180 minutes (mean ± SEM; 0.75 ± 0.12 mmol/L [baseline] vs 1.45 ± 0.26 mmol/L) post‐OFTT, respectively.

There was a significant difference in the postprandial factor H response between PCOS and control groups (Figure 2B; Table S5). In controls, factor H levels fell sharply from baseline during the first hour post‐OFTT (mean ± SEM; 250.9 ± 21.49 µg/mL vs 173.6 ± 15.26 µg/mL). In contrast, in the PCOS group, factor H levels increased 30 minutes post‐OFTT. Consequently, the AUC for factor H was significantly different between PCOS and controls (Table S5).

Postprandial TCC, C3 and C4 (Figure 2C‐E), but not properdin or C3a(desArg; Figure 2F‐G), levels increased from baseline in both controls and PCOS subjects, but no differences were observed between groups (Table S5).

### 3.5 Effect of obesity on the complement response to OFTT

**Figure 3:**
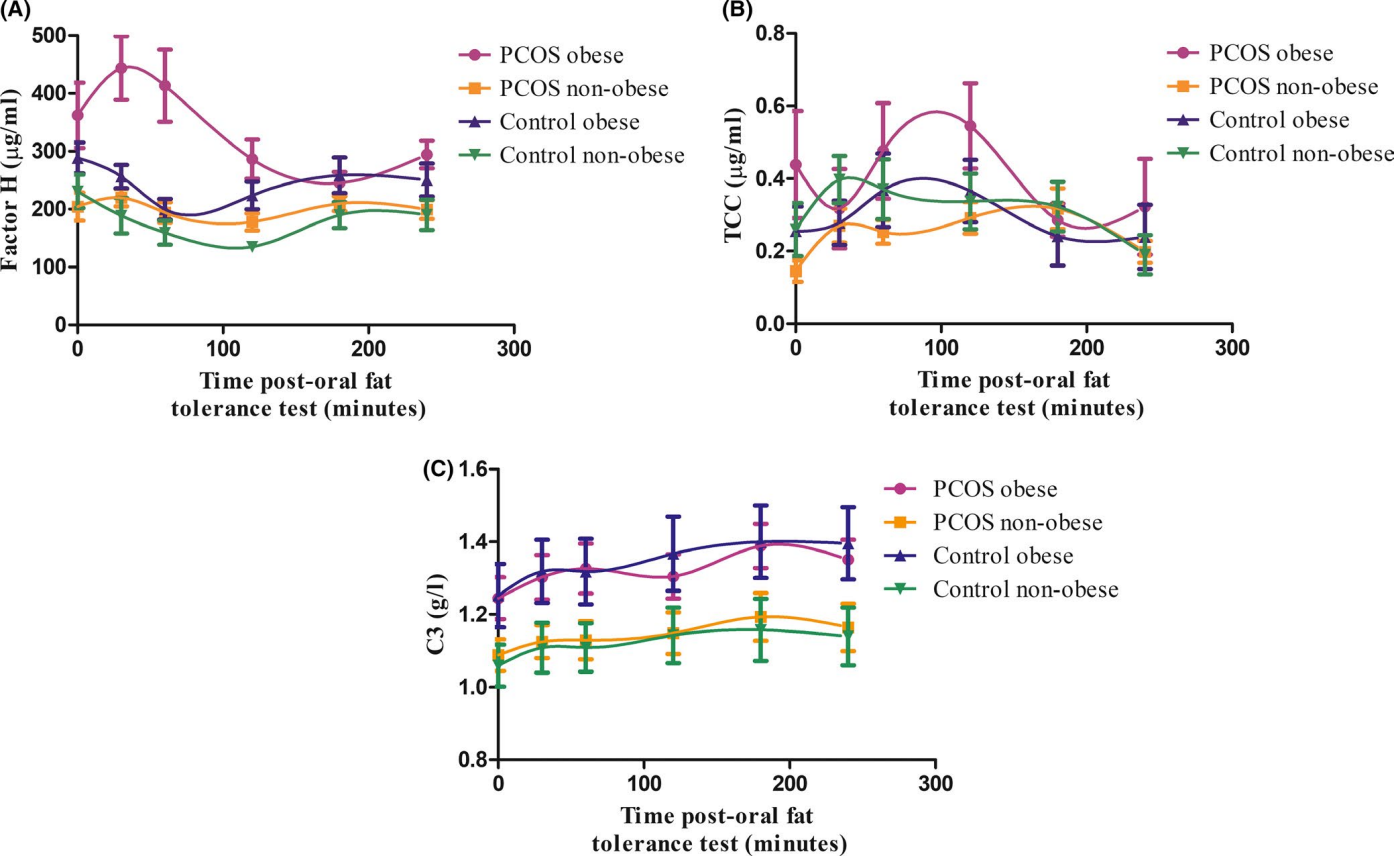
Influence of obesity on factor H, C3 and TCC responses to postprandial lipaemia. Postprandial factor H (A), TCC (B) and C3 (C) responses to OFTT in obese (BMI ≥ 30 kg/m^2^) and non‐obese (BMI < 30 kg/m^2^) PCOS and control subjects (cohort 2). Obese and non‐obese controls. n = 20 PCOS, n = 20 Controls

**Figure 4:**
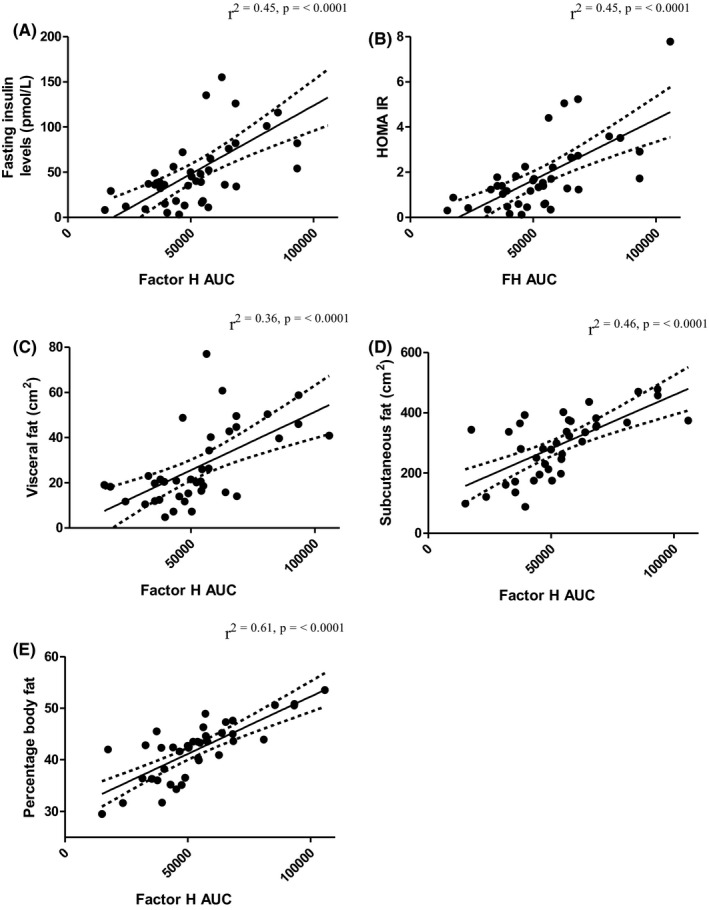
Relationship between factor H area under the curve (AUC), body fat and metabolic parameters. Data represent PCOS subjects and controls in cohort 2 (n = 40)

**Figure 5:**
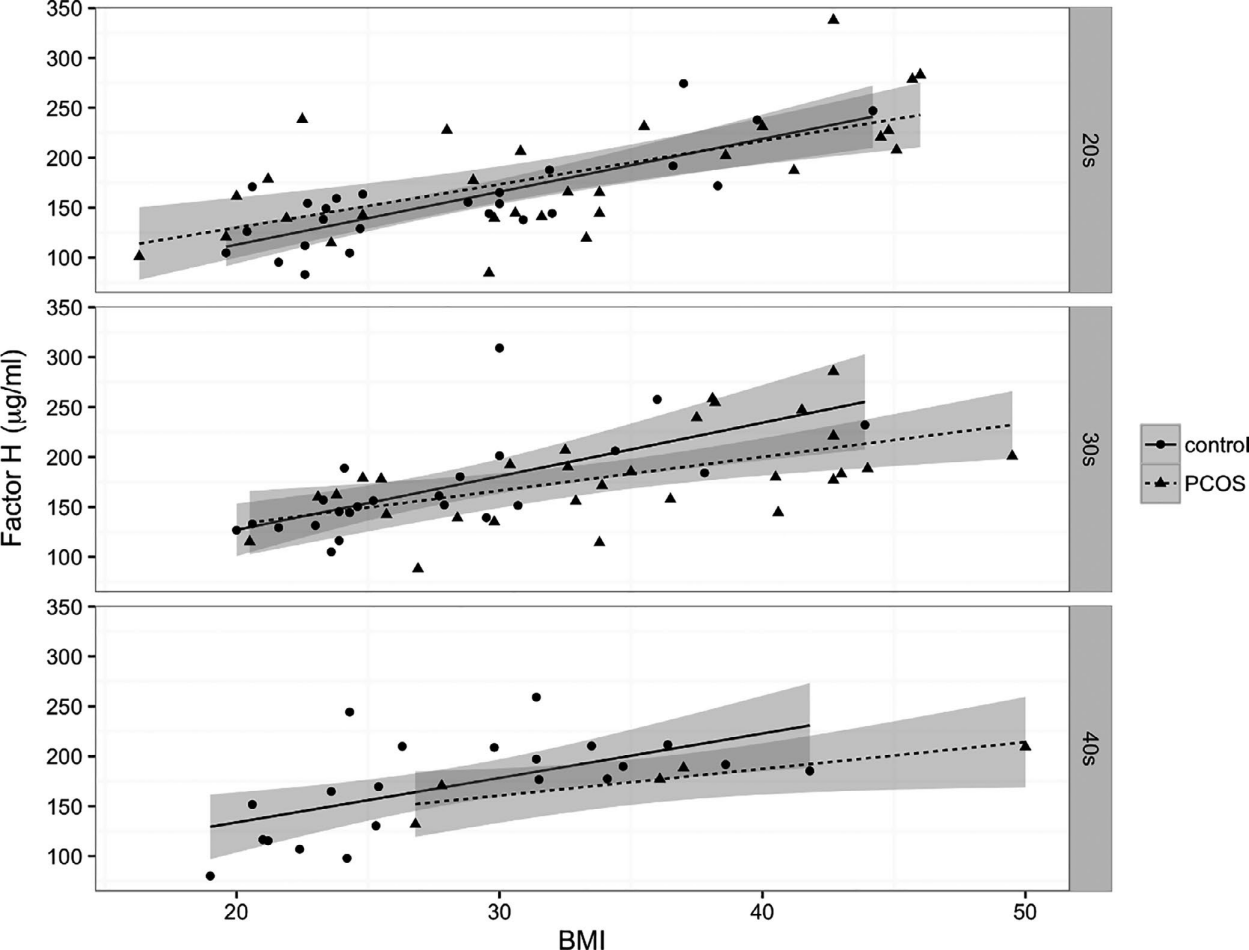
Correlation between fasting factor H concentrations and BMI. Data represent correlations between factor H levels and BMI in different age groups in PCOS subjects (triangles, dashed line) and controls (circles, solid line; cohort 1). Factor H levels correlated positively with BMI in both PCOS (n = 84) and controls (n = 95) in all age groups

We subsequently divided the PCOS and control groups into ‘obese’ (BMI ≥ 30 kg/m^2^) and ‘non‐obese’ (BMI < 30 kg/m^2^) groups and compared the effects of lipaemia on plasma levels of factor H, TCC and C3 in these groups (Figure 3A‐C, respectively). Within the PCOS group, obesity had a profound effect on both baseline levels and AUC for factor H. Fasting factor H levels and AUC were both markedly increased in obese vs non‐obese PCOS subjects (mean ± SEM; baseline: 362.1 ± 56.41 µg/mL vs 204.3 ± 24.05 µg/mL, respectively; *P* < .001, and AUC: 1717 ± 164.1 µg min/mL vs 1004 ± 54.4 µg min/mL, respectively; *P* < .0001). Furthermore, across the whole cohort, strong correlations were observed between factor H AUC and each of HOMA‐IR, visceral and subcutaneous fat area (Figure 4; *P* < .0001 for all), and in fasting samples (cohort 1) between factor H and BMI, regardless of age (Figure 5; age 20‐30: *r* ^2^ .43, *P* < .001 [PCOS] *r* ^2^ .62, *P* < .001 [controls]; age 30‐40: *r* ^2^ .30, *P* < .001 [PCOS] *r* ^2^ .44, *P* < .001 [controls]; age 40‐50: *r* ^2^ .77, *P* < .05 [PCOS], *r* ^2^ .36, *P* < .001 [controls]). There was no evidence of a relationship between factor H AUC and testosterone (data not shown). Baseline C3 levels were increased in obese compared to non‐obese subjects irrespective of disease status, with C3 concentrations increasing gradually across the time course of the OFTT in both groups (Figure 3C).

## 4 DISCUSSION

In this analysis of complement proteins and activation products in young women, we find evidence of complement dysregulation in patients with PCOS (Figure S2). Activation of the complement cascade was evident in the fasting state as well as postprandially and involved both the activation (C3, C3(adesArg), factor H) and terminal pathways (C5a(desArg), TCC). Furthermore, complement dysregulation was most pronounced in metabolically unhealthy subjects and correlated with both obesity and insulin sensitivity.

Our findings are consistent with some^17^, ^18^, ^19^ but not all^20^, ^21^ previous studies in which C3 and C3a(desArg) levels were found to be increased in women with PCOS compared to controls. C3a(desArg), also known as acylation‐stimulating protein (ASP), increases energy storage through a number of actions, including enhanced glucose uptake, reduced lipolysis and increased triglyceride clearance.^27^ Consistent with these observations, C3a(desArg) and C3 levels are increased in subjects with obesity and type 2 diabetes,^28^, ^29^ whilst C3 knockout mice display reduced body weight and fat mass.^27^ Longitudinal human data have additionally shown that changes in C3 levels are positively associated with increase in BMI,^30^ incident obesity^31^ and metabolic syndrome, implying a possible role for complement dysregulation in human obesity.

C3a(desArg) is synthesized in a two‐step process involving C3, factor B and factor D; cleavage of the parent molecule C3 generates C3a, which subsequently undergoes desargination of the carboxyl terminus to generate C3a(desArg). In contrast to C3, fasting levels of factor B and factor D were only elevated in our PCOS subjects in unadjusted analyses, suggesting that obesity was the main factor accounting for these increases. This accords with similar findings from Daan *et al*,^32^ who found no difference in factor D levels in either hyperandrogenic or normoandrogenic PCOS subjects compared with unaffected controls, and with other studies showing elevated factor B and D concentrations in obesity.^28^, ^33^, ^34^ In contrast, Gursoy Calan *et al* ^16^ found that factor D levels were elevated in women with PCOS compared with age‐ and BMI‐matched controls and that circulating concentrations correlated with both BMI and HOMA‐IR.

Properdin and factor H are important regulators of alternative pathway activation: properdin acts as a stabilizer of C3 convertase leading to prolonged complement activation, whilst factor H functions as an inhibitor by dissociating C3 convertase; elevations in both reflect alternative pathway dysregulation. Whilst fasting properdin concentrations were not different in our unadjusted analyses, properdin levels were marginally lower after adjustment for BMI and age. In contrast, fasting factor H concentrations were higher in PCOS subjects only before adjustment for the group differences in BMI. This observation is consistent with previous cross‐sectional and longitudinal studies which have shown positive associations between factor H concentrations and adiposity.^30^, ^35^ We also found elevated TCC concentrations in women with PCOS, indicating increased terminal pathway activation. Increased TCC levels are likely caused by alternative pathway dysregulation since inhibition of the alternative pathway reduces TCC concentrations by >80%.^36^, ^37^

In view of the differences we observed in fasting complement protein levels of both activation and terminal pathways, we subsequently sought to compare the kinetics of complement protein generation in response to a fatty meal. We were keen to focus on the impact of lipaemia since postprandial triglycerides predict cardiovascular risk better than fasting levels,^38^ not least because humans exist in a postprandial state for most of the day. Lipaemic challenge resulted in a substantial increase in triglyceride levels whilst C3, C4 and TCC increased to a similar extent in both groups. In contrast, whilst factor H levels fell in controls, an acute rise was observed in PCOS subjects, notably in the presence of co‐existing obesity. Several complement components have been implicated in lipid metabolism. Chylomicrons, which transport dietary lipids, can spontaneously activate the alternative pathway and become coated in C3 opsonic fragments.^11^ C3 fragments are also present in other lipoproteins, including apolipoprotein‐A1 (which associates with chylomicrons), and in HDL.^39^ Factor H regulates chylomicron‐induced C3 activation during lipid storage^11^ and alternative pathway complement activation on HDL.^40^ Obesity appeared to be a significant driver for the elevated factor H levels in our PCOS subjects in both the fasting and postprandial state. Insulin resistance also correlated significantly with postprandial factor H area under the curve. Since adiposity and insulin resistance are intimately linked, it is difficult to establish which of these parameters is the dominant driver. However, it is interesting to note that not only does insulin resistance correlate with omental fat factor H expression^35^ but also that addition of factor H to freshly isolated pancreatic islet cells reduces insulin secretion.^41^ It is thus conceivable that a bidirectional effect may exist, whereby reduced insulin sensitivity is both a mediator and consequence of alternative pathway complement activation.

In contrast to C3, postprandial C3a(desArg) levels did not alter from baseline. At first glance, this may seem surprising given the role of C3a(desArg) in increasing lipid uptake and storage. However, other studies have also shown unaltered circulating C3a(desArg) levels in response to a fat meal.^12^, ^42^ This may be attributable to local as opposed to systemic C3a(desArg) formation and rapid adipocyte uptake upon subsequent C5L2 receptor binding. In line with this, veno‐arterial gradient studies have confirmed that C3a(desArg) is generated in vivo by human adipocytes and that this production is exaggerated postprandially, especially in the presence of obesity.^43^, ^44^ Indeed, adipocytes are an important source of complement components, particularly those of the alternative pathway. Both visceral and subcutaneous adipose tissue are known to produce and secrete C3, C4, factor D, properdin and factor H, creating an environment for local generation of C3a(desArg).^29^, ^35^, ^45^

In light of our findings of complement dysregulation in women with PCOS, it would be interesting to explore whether therapeutic strategies to reduce alternative pathway activation might alleviate or prevent complications associated with PCOS such as metabolic syndrome, type 2 diabetes and cardiovascular disease. In this context, it is interesting to note that C3a(desArg) levels are reduced by weight loss^46^ and physical activity^47^ in obesity, whilst C3 and C3a(desArg) levels fall in response to metformin therapy in women with PCOS.^18^ Statins have also been shown to reduce C3 and C3a(desArg) levels in patients with cardiovascular risk, both in the fasting and postprandial state.^15^, ^48^, ^49^ Additional studies examining the effects of new compounds that selectively reduce alternative complement pathway activation^50^ would be of therapeutic interest, not only in PCOS but also in other cardiometabolic disorders associated with enhanced complement activation. However, any such treatments would need a careful evaluation of safety, especially with regard to fertility and pregnancy, in this young, reproductive age population.

Our study has both strengths and limitations. To our knowledge, this is the most comprehensive analysis to date of the complement system in patients with PCOS, benefiting from careful anthropometric and metabolic characterization in addition to measurements in the postprandial as well as the fasting state. Nevertheless, whilst we intentionally sought to compare postprandial complement activation kinetics in a carefully matched population of PCOS subjects and controls, our study was limited by the absence of an additional group of insulin‐resistant women with PCOS. We were also limited to a 4‐hour study window after the fat challenge.

In summary, we demonstrate evidence of complement activation and dysregulation in women with PCOS which is exacerbated in the presence of obesity and insulin resistance. We show that this extends to the terminal pathway and is evident in the postprandial as well as the fasting state. These disturbances have implications for lipid clearance, inflammation and insulin sensitivity and suggest that studies are needed to explore whether interventions aimed at regulating complement activation in PCOS may be helpful in reducing cardiometabolic risk.

## CONFLICT OF INTEREST

The authors have no conflicts of interest to declare.

## Supporting information

Figure S1

Figure S2

Tables S1‐S5

Supplementary Material

## Data Availability

The data that support the findings of this study are available from the corresponding author upon reasonable request.
