# Supplementary material for: Complement activation in polycystic ovary syndrome occurs in the postprandial and fasted state and is influenced by obesity and insulin sensitivity
Source: Clin Endocrinol (Oxf). 2020 Sep 15;94(1):74–84. doi: 10.1111/cen.14322 (PMC9623543; doi:10.1111/cen.14322)
Supplement: Supplementary file 1 — Figure S1 [file CEN-94-74-s003.pptx]

## Slide 1
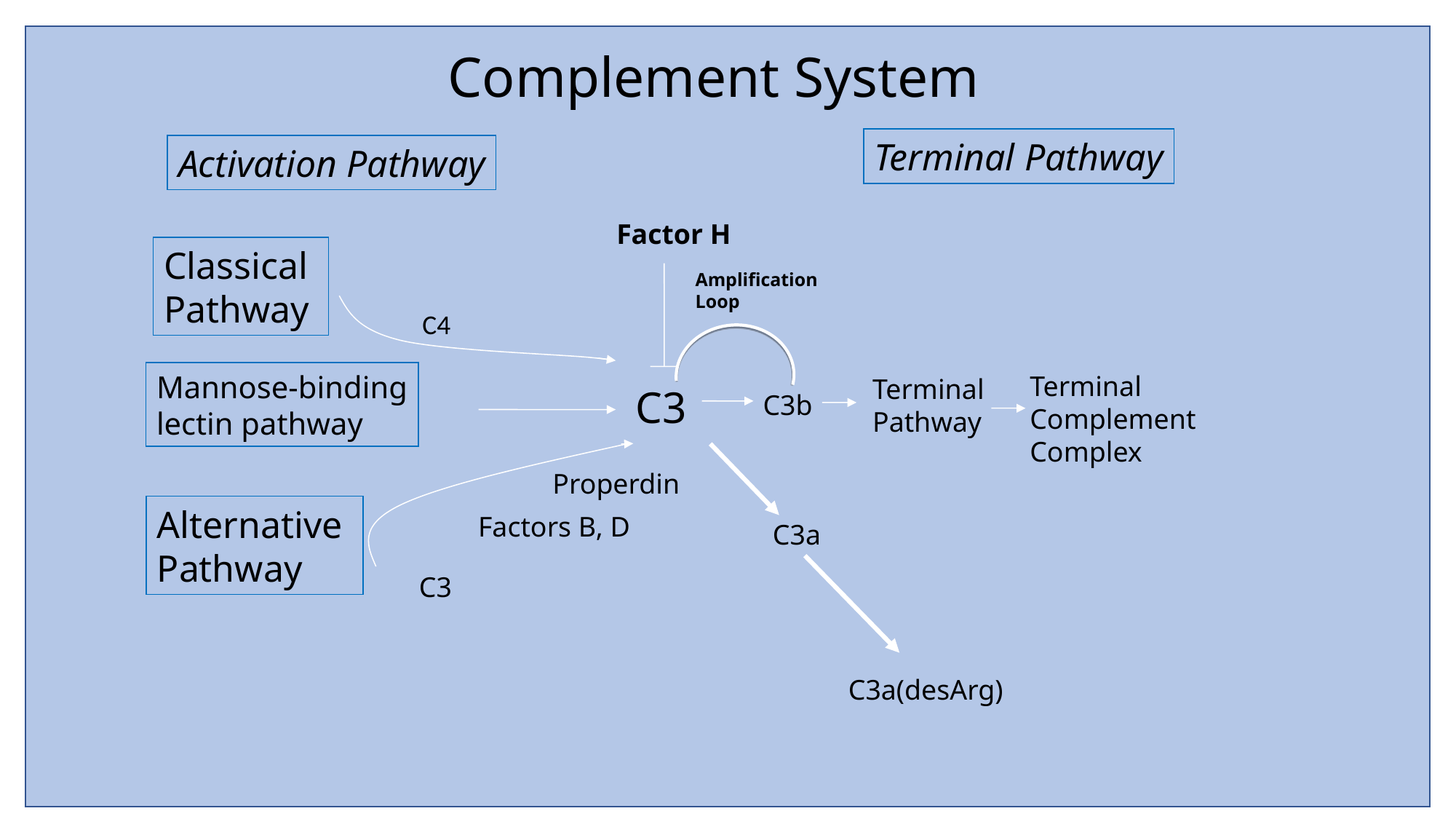

# Complement System
Terminal Pathway
Activation Pathway
Factor H
Classical
Pathway
Amplification
Loop
C4
Mannose-binding
lectin pathway
Terminal
Complement
Complex
Terminal
Pathway
C3
C3b
Properdin
Alternative
Pathway
Factors B, D
C3a
C3
C3a(desArg)
