# Supplementary material for: Complement activation in polycystic ovary syndrome occurs in the postprandial and fasted state and is influenced by obesity and insulin sensitivity
Source: Clin Endocrinol (Oxf). 2020 Sep 15;94(1):74–84. doi: 10.1111/cen.14322 (PMC9623543; doi:10.1111/cen.14322)
Supplement: Supplementary file 2 — Figure S2 [file CEN-94-74-s002.pptx]

## Slide 1
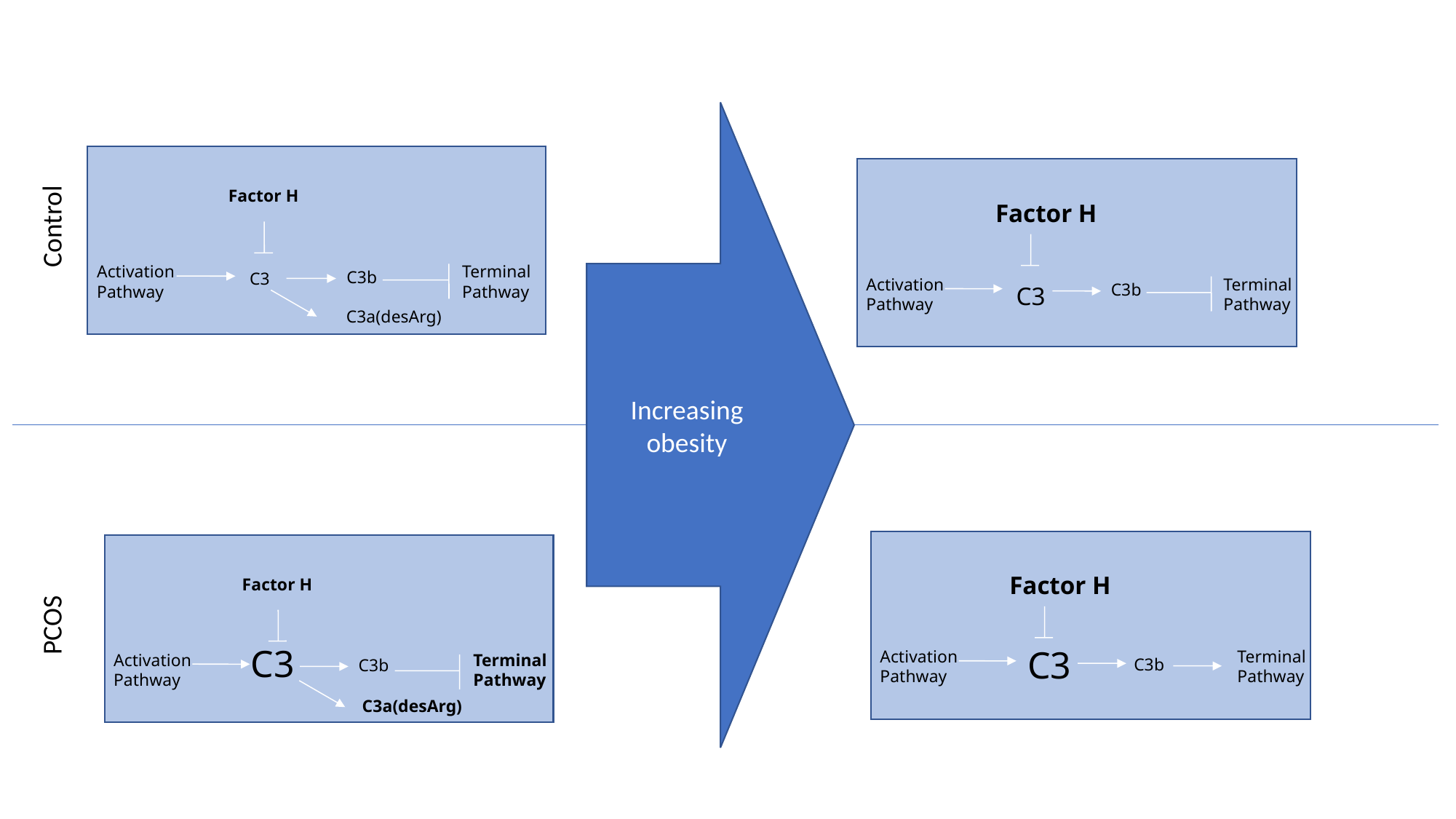

Factor H
C3
Activation
Pathway
Terminal
Pathway
C3b
Increasing obesity
Factor H
Activation
Pathway
Terminal
Pathway
C3b
C3
Factor H
Control
Activation
Pathway
Terminal
Pathway
C3b
C3
C3a(desArg)
Factor H
C3
Activation
Pathway
Terminal
Pathway
C3b
PCOS
C3a(desArg)
