# Supplementary material for: Complement activation in polycystic ovary syndrome occurs in the postprandial and fasted state and is influenced by obesity and insulin sensitivity
Source: Clin Endocrinol (Oxf). 2020 Sep 15;94(1):74–84. doi: 10.1111/cen.14322 (PMC9623543; doi:10.1111/cen.14322)
Supplement: Supplementary file 3 — Tables S1‐S5 [file CEN-94-74-s004.docx]

**Table S1.** Clinical characteristics of the subjects in cohort 1.

|  | | |  | **Unadjusted** | | |  | **Adjusted for age and BMI** | | |
| --- | --- | --- | --- | --- | --- | --- | --- | --- | --- | --- |
|  | **PCOS (n=84)** | **Control (n=95)** | **2.5% CI** | **Difference (PCOS-control)** | **97.5% CI** | **p-value** | **2.5% CI** | **Difference (PCOS-control)** | **95% CI** | **p-value** |
| Age (years) | 29.8 (6.7) | 32.6 (7.9) | -5.0 | -2.9 | -0.7 | 0.01 |  |  |  |  |
| BMI (kg/m^2^) | 33.3 (7.8) | 27.6 (6.3) | 3.6 | 5.7 | 7.8 | <0.001 |  |  |  |  |
| Total cholesterol (mmol/l) | 4.80 (0.75) | 4.63 (0.83) | -0.06 | 0.17 | 0.41 | 0.152 | -0.06 | 0.19 | 0.45 | 0.139 |
| HDL cholesterol (mmol/l) | 1.32 (0.35) | 1.39 (0.33) | -0.17 | -0.07 | 0.03 | 0.166 | -0.04 | 0.07 | 0.17 | 0.196 |
| LDL cholesterol (mmol/l) | 2.97 (0.68) | 2.79 (0.78) | -0.04 | 0.18 | 0.40 | 0.115 | -0.13 | 0.11 | 0.35 | 0.346 |
| Triglycerides (mmol/l) | 1.11 (0.47) | 0.96 (0.45) | 0.01 | 0.15 | 0.29 | 0.031 | -0.13 | 0.01 | 0.16 | 0.841 |
| HOMA-IR | 4.94 (4.15) | 2.33 (1.45) | 1.71 | 2.61 | 3.52 | <0.001 | 0.45 | 1.33 | 2.21 | 0.003 |
| hs-CRP (mg/l) | 4.67 (5.92) | 2.49 (3.04) | 0.81 | 2.18 | 3.55 | 0.002 | -0.95 | 0.41 | 1.78 | 0.553 |
| Testosterone (nmol/l) | 1.39 (0.81) | 0.77 (0.38) | 0.43 | 0.61 | 0.80 | <0.001 | 0.36 | 0.57 | 0.78 | <0.001 |
| Total fat area (cm^2^) | 361 (126) | 286 (130) | 30.6 | 75 | 110.8 | 0.001 | -27.3 | -1.6 | 24.1 | 0.904 |
| Subcutaneous fat area (cm^2^) | 322 (110) | 257 (115) | 25.2 | 65 | 96.1 | 0.001 | -27.7 | -3.4 | 20.9 | 0.783 |
| Visceral fat area (cm^2^) | 39.1 (28.2) | 28.5 (19.5) | 2.7 | 10.6 | 17.4 | 0.007 | -3.9 | 1.8 | 7.5 | 0.529 |

Unadjusted and age/BMI-adjusted anthropometric and metabolic characteristics of the study population. CI: confidence interval, HOMA-IR: homeostasis model assessment of insulin resistance, hs-CRP: high sensitivity c-reactive protein. Data are presented as means (± standard deviation). n=84 PCOS, n=95 Controls.

**Table S2.** Correlation of complement components with measures of adiposity, lipids and HOMA-IR.

|  | **BMI** | **WHR** | **Visceral fat area** | **SC fat area** | **Total cholesterol** | **HDL cholesterol** | **LDL cholesterol** | **Triglycerides** | **HOMA-IR** |
| --- | --- | --- | --- | --- | --- | --- | --- | --- | --- |
| **C4** | 0.552*** | 0.205* | 0.386*** | 0.471*** | 0.158 | -0.266* | 0.206* | 0.417*** | 0.306*** |
| **C3** | 0.590*** | 0.256* | 0.507*** | 0.554*** | 0.296* | -0.280* | 0.334** | 0.504*** | 0.448*** |
| **C3adesArg** | -0.016 | 0.034 | 0.119 | -0.004 | -0.010 | 0.096 | -0.104 | 0.135 | 0.095 |
| **C3a/C3** | -0.113 | -0.040 | -0.033 | -0.144 | -0.105 | 0.148 | -0.223* | 0.084 | -0.011 |
| **Factor B** | 0.403*** | 0.195* | 0.308* | 0.296* | 0.260* | -0.179 | 0.333** | 0.340** | 0.255* |
| **Factor H** | 0.614*** | 0.370*** | 0.598*** | 0.511*** | 0.144 | -0.328*** | 0.245* | 0.315** | 0.548*** |
| **Properdin** | 0.545*** | 0.325** | 0.455*** | 0.600*** | 0.140 | -0.328*** | 0.240* | 0.272* | 0.403*** |
| **Factor D** | 0.317** | 0.153 | 0.116 | 0.185* | -0.059 | -0.177* | -0.006 | 0.119 | 0.074 |
| **C5a** | -0.116 | -0.042 | -0.125 | -0.102 | 0.020 | 0.065 | 0.091 | 0.284* | -0.161 |
| **C5** | 0.285* | 0.194* | 0.362** | 0.236* | 0.245* | -0.151 | 0.285* | 0.308* | 0.260* |
| **TCC** | 0.045 | 0.218* | 0.246* | 0.116 | 0.114 | 0.001 | 0.078 | 0.177* | 0.098 |

WHR: Waist:hip ratio, SC: subcutaneous, HDL: High density lipoprotein cholesterol, LDL: Low density lipoprotein cholesterol, HOMA-IR: Homeostasis model assessment of insulin resistance, TCC: terminal complement complex. *p<0.05; **p<0.01; ***p<0.001. n=84 PCOS, n=95 Controls.

**Table S3.** Clinical characteristics of the subjects in cohort 2

|  | | | **Unadjusted** | | | | **Adjusted for age, BMI and smoking** | | | |
| --- | --- | --- | --- | --- | --- | --- | --- | --- | --- | --- |
|  | **PCOS** | **Control** | **2.5% CI** | **Difference (PCOS – control)** | **97.5% CI** | **p-value** | **2.5% CI** | **Difference (PCOS – control)** | **97.5% CI** | **p-value** |
| Age (years) | 29.8 (7.8) | 30.9 (7.7) | -6.0 | -1.1 | 3.8 | 0.65 |  |  |  |  |
| BMI (kg/m^2^) | 29.0 (6.8) | 28.1 (4.2) | -2.7 | 0.9 | 4.5 | 0.63 |  |  |  |  |
| Total cholesterol (mmol/l) | 3.95 (1.03) | 3.9 (1.36) | -0.72 | 0.06 | 0.83 | 0.89 | -0.53 | 0.16 | 0.85 | 0.64 |
| HDL cholesterol (mmol/l) | 1.21 (0.41) | 1.19 (0.48) | -0.26 | 0.02 | 0.31 | 0.88 | -0.18 | 0.08 | 0.34 | 0.54 |
| LDL cholesterol (mmol/l) | 2.57 (0.69) | 2.56 (1.01) | -0.55 | 0.01 | 0.57 | 0.97 | -0.43 | 0.08 | 0.59 | 0.75 |
| Triglyceride (mmol/l) | 0.86 (0.58) | 0.74 (0.52) | -0.23 | 0.12 | 0.47 | 0.5 | -0.2 | 0.15 | 0.49 | 0.4 |
| Triglyceride AUC (mmol min/l) | 302 (191) | 283 (205) | -108 | 19 | 145 | 0.77 | -114 | 11 | 137 | 0.86 |
| Glucose (mmol/l) | 4.65 (0.34) | 4.61 (0.37) | -0.18 | 0.04 | 0.26 | 0.72 | -0.14 | 0.06 | 0.27 | 0.54 |
| Glucose AUC (mmol min/l) | 1063 (76) | 1084 (81) | -72 | -21 | 29 | 0.38 | -69 | -23 | 24 | 0.34 |
| Insulin (pmol/l) | 61.05 (51.02) | 43.7 (37.12) | -11.21 | 17.35 | 45.91 | 0.23 | -8.68 | 12.62 | 33.92 | 0.24 |
| Insulin AUC (pmol min/l) | 45 (49) | 33 (40) | -16 | 12 | 41 | 0.38 | -17 | 8 | 32 | 0.54 |
| HOMA-IR | 2.09 (1.8) | 1.51 (1.36) | -0.44 | 0.58 | 1.61 | 0.25 | -0.34 | 0.41 | 1.16 | 0.27 |
| Testosterone (nmol/l) | 1.47 (0.61) | 1.13 (0.41) | <0.0001 | 0.34 | 0.68 | 0.05 | -0.01 | 0.32 | 0.65 | 0.06 |
| Visceral fat area (cm^2^) | 28 (17) | 26 (18) | -10 | 2 | 13 | 0.77 | -10 | 0.2 | 10 | 0.97 |
| Total fat area (cm^2^) | 320 (119) | 320 (116) | -76 | 0.45 | 77 | 0.99 | -65 | -13 | 38 | 0.6 |
| Subcutaneous fat area (cm^2^) | 292 (105) | 293 (108) | -70 | -1 | 68 | 0.97 | -60 | -14 | 33 | 0.56 |

Unadjusted and age, BMI and smoking-adjusted anthropometric and metabolic characteristics of the study population. CI: confidence interval, HDL: High density lipoprotein, LDL: Low density lipoprotein, AUC: area under the curve, HOMA-IR: homeostasis model assessment of insulin resistance. Data are presented as means (± standard deviation). n=20 PCOS, n=20 Controls.

**Table S4.** Fasting plasma complement levels in cohort 2

|  |  |  | | | **Unadjusted** | | | | | | **Adjusted for age, BMI and smoking** | | | | | | | | |
| --- | --- | --- | --- | --- | --- | --- | --- | --- | --- | --- | --- | --- | --- | --- | --- | --- | --- | --- | --- |
|  | **PCOS** | **Control** | | **2.5% CI** | | **Difference (PCOS – control)** | | **97.5% CI** | | **p - value** | | **2.5% CI** | **Difference (PCOS – control)** | | | **97.5% CI** | | **p - value** | |
|  |  |  | |  | |  | |  | |  |  | |  | |  | |  | |  |
| C4 (g/l) | 0.21 (0.07) | | 0.21 (0.05) | -0.04 | | 0 | | 0.04 | | 0.88 | -0.03 | | 0.01 | | 0.04 | | 0.73 | |  |
| C3 (g/l) | 1.15 (0.17) | | 1.13 (0.23) | -0.11 | | 0.02 | | 0.15 | | 0.71 | -0.09 | | 0.02 | | 0.13 | | 0.74 | |  |
| C3a(desArg) (ng/ml) | 89.22 (48.46) | | 111.52 (65.65) | -59.24 | | -22.3 | | 14.63 | | 0.23 | -59.24 | | -22.3 | | 14.63 | | 0.23 | |  |
| C3a/C3 ratio | 79.35 (44.08) | | 102.54 (61.19) | -57.33 | | -23.19 | | 10.95 | | 0.18 | -54.4 | | -20.95 | | 12.5 | | 0.21 | |  |
| Factor H (µg/ml) | 267.41 (140.32) | | 250.94 (96.09) | -60.52 | | 16.47 | | 93.45 | | 0.67 | -61.13 | | 7.38 | | 75.88 | | 0.83 | |  |
| Properdin (µg/ml) | 10703.26 (3351.5) | | 10861.05 (4073.3) | -2585.22 | | -157.79 | | 2269.65 | | 0.9 | -2585.22 | | -157.79 | | 2269.65 | | 0.9 | |  |
| TCC (µg/ml) | 0.26 (0.27) | | 0.26 (0.21) | -0.17 | | 0.01 | | 0.18 | | 0.95 | -0.18 | | -0.01 | | 0.16 | | 0.91 | |  |
|  |  |  | | |  | |  | |  |  |  | | |  | |  | |  | |

Data are presented as means (± standard deviation). CI: confidence interval, TCC: Terminal complement complex. n=20 PCOS, n=20 Controls.

**Table S5.** Post-oral fat tolerance test complement measurements in cohort 2.

|  |  |  |  | | **Unadjusted** | | | | **Adjusted for age, BMI and smoking** | | | |
| --- | --- | --- | --- | --- | --- | --- | --- | --- | --- | --- | --- | --- |
|  |  | **PCOS** | **Control** | | **2.5%** | **Difference (PCOS – control)** | **97.5%** | **p - value** | **2.5%** | **Difference (PCOS – control)** | **97.5%** | **p - value** |
| Area under the curve | C4 (g min/l) | 54.2  (17.2) | 54.2  (15.6) | -10.51 | | 0.01 | 10.52 | 1 | -9.02 | 0.79 | 10.6 | 0.87 |
|  | C3 (g min/l) | 293.63  (48.17) | 290.24  (66.78) | -33.88 | | 3.4 | 40.67 | 0.85 | -30.04 | 1.25 | 32.53 | 0.94 |
|  | C3a(desArg)  (ng min/ml) | 22866.3  (12071.9) | 32911.9  (36029.4) | -27246.1 | | -10045.7 | 7154.8 | 0.24 | -27246.1 | -10045.7 | 7154.8 | 0.24 |
|  | C3a/C3 ratio | 19443.05  (10090.0) | 28040.6  (30301.3) | -23054.4 | | -8597.55 | 5859.34 | 0.24 | -22161.9 | -7817.34 | 6527.26 | 0.28 |
|  | Factor H  (µg min/ml) | 59819.3  (20728.38) | 45990.55  (16826.69) | 1743.24 | | 13828.75 | 25914.26 | 0.03 | 4942.44 | 12167.31 | 19392.18 | <0.001 |
|  | Properdin  (µg min/ml) | 2536.95  (746.68) | 2631.35  (735.87) | -575.49 | | -94.4 | 386.69 | 0.69 | -575.49 | -94.4 | 386.69 | 0.69 |
|  | TCC (µg min/ml) | 68.67 (41.11) | 69.74 (41.57) | -29.08 | | -1.08 | 26.93 | 0.94 | -29.08 | -1.08 | 26.93 | 0.94 |

Data are means (± standard deviation). TCC: Terminal complement complex. n=20 PCOS, n=20 Controls.
