# Supplementary material for: Complement activation in polycystic ovary syndrome occurs in the postprandial and fasted state and is influenced by obesity and insulin sensitivity
Source: Clin Endocrinol (Oxf). 2020 Sep 15;94(1):74–84. doi: 10.1111/cen.14322 (PMC9623543; doi:10.1111/cen.14322)
Supplement: Supplementary file 4 — Supplementary Material [file CEN-94-74-s001.docx]

**Figure S1.** Complement activation cascade. The activation pathway consists of classical, mannose-binding and the alternative pathway. Activation of all early pathways of complement lead to C3 activation, which in turn, results in terminal pathway activation. Activation of C3 can also lead to the activation of the terminal pathway via C5, leading to the formation of the pore like structure, terminal complement complex (TCC). Activation and terminal pathways of complement are tightly regulated by complement regulators. For instance, the alternative pathway of complement, activated by spontaneous hydrolysis of C3 is dependent on the presence of factor B, D and Properdin and is tightly regulated by proteins such as factor H.

**Figure S2.** Activation and terminal complement pathway in patients with PCOS and controls. Insulin-resistant subjects with PCOS show elevation of fasting plasma C3, C3a(desArg) and terminal complement complex compared to healthy controls. Obesity increases C3 in both groups, but to a greater extent in patients with PCOS. Factor H, a regulator of alternative pathway complement activation, is also increased in obesity, especially in patients with PCOS postlipaemia.
